# Evaluation of Clinical Outcome after Laparoscopic Antireflux Surgery in Clinical Practice: Still a Controversial Issue

**DOI:** 10.1155/2011/725472

**Published:** 2011-09-11

**Authors:** Sandro Contini, Carmelo Scarpignato

**Affiliations:** ^1^Department of Surgical Sciences, University of Parma, 43125 Parma, Italy; ^2^Laboratory of Clinical Pharmacology, Division of Gastroenterology, Department of Clinical Sciences, School of Medicine & Dentistry, University of Parma, 43125 Parma, Italy

## Abstract

*Background*. Laparoscopic antireflux surgery has shown to be effective in controlling gastroesophageal reflux (GERD). Yet, a universally accepted definition and evaluation for treatment success/failure in GERD is still controversial. The purpose of this paper is to assess if and how the outcome variables used in the different studies could possibly lead to an homogeneous appraisal of the limits and indications of LARS. *Methods*. We analyzed papers focusing on the efficacy and outcome of LARS and published in English literature over the last 10 years. *Results*. Symptoms scores and outcome variables reported are dissimilar and not uniform. The most consistent parameter was patient's satisfaction (mean satisfaction rate: 88.9%). Antireflux medications are not a trustworthy outcome index. Endoscopy and esophageal manometry do not appear very helpful. Twenty-four hours pH metry is recommended in patients difficult to manage for recurrent typical symptoms. *Conclusions*. More uniform symptoms scales and quality of life tools are needed for assessing the clinical outcome after laparoscopic antireflux surgery. In an era of cost containment, objective evaluation tests should be more specifically addressed. Relying on patient's satisfaction may be ambiguous, yet from this study it can be considered a practical and simple tool.

## 1. Introduction

Laparoscopic antireflux surgery (LARS) has shown to be effective in controlling gastroesophageal reflux [1, 2]. However, a universally accepted definition for treatment success/failure in gastroesophageal reflux disease (GERD) is not yet available: objective evaluation of symptoms, response to treatment, and definition of treatment failure are all still controversial. A substantial number of the patients after surgery still take antireflux medications (ARMs) [3–5], with percentages ranging from 62% to 15–20% after 9 and 4-5 years of followup, respectively [6–12]. ARM use is performed on the assumption that foregut symptoms in a patient after fundoplication are consequent to a failed operation and based on the assumption that a diagnosis of recurrent reflux can be made confidently from the clinical findings [13, 14]. However, most patients taking acid suppressive medications after antireflux surgery do not reveal any abnormal esophageal acid exposure [15], and the presence of symptoms alone may not seem to be a good reason to start an antacid treatment. Therefore, the prescription of ARM frequently seems inappropriate and does not always indicate that surgical therapy has failed.

Reports dealing with the clinical outcome after LARS, either concentrate on symptomatic results, patient's satisfaction, and quality of life, on the percentage of patients taking ARM, or on the objective evaluation of the esophageal function and acid exposure. Yet, there is not agreement on how should a successful outcome be defined and how could the consequent therapeutic approach be directed.

On this background, we felt worthwhile to analyze the recent literature, mainly focused on the efficacy and outcome of LARS. The purpose of this paper is therefore

- to assess if and how the outcome variables used in the different studies could possibly lead, in spite of their complexity, to an homogeneous appraisal of the limits and indications of LARS in the management of GERD,
- how these outcome evaluations could be better interpreted in order to identify failures of treatment,
- to possibly extrapolate and suggest a flowchart for the postoperative evaluation after LARS.

## 2. Methods

In order to evaluate criteria and definition of a successful clinical outcome after LARS, we analyzed studies, published after 2000, which were specifically performed to assess reflux symptoms, medication assumption, satisfaction to surgery, evaluation of quality of life, and objective esophageal tests after LARS. Specifically, for each study, it was taken into account:

- parameters utilized to assess the clinical outcome, that is, clinical evaluation or interview, phone interview, symptoms questionnaire or others (QoL, GIQLI, HRQL, GSRS, PGWB), analysis of hospital data bases, level of satisfaction;
- incidence of GERD and not GERD-related symptoms;
- use of ARM (either continuous or occasional) for GERD-related and not GERD symptoms;
- response to medications and, when specified, the main prescriptor (family physician, gastroenterologist, surgeon);
- objective esophageal tests (endoscopy, esophageal manometry, 24-hour esophageal pH-metry) when performed.

## 3. Results

Thirty-four papers [2–6, 8, 11, 14–40] concerning clinical outcome after LARS were evaluated. The total number of patients included in this review was 7599, with a follow-up ranging from a minimum of 6 months to 12 years. The first author was a gastroenterologist in 7 (21.8%) papers and a surgeon in 26. Twenty-five studies came from highly specialized or university hospitals, 2 from VA cooperative studies [6, 26], 3 from cooperative studies between university hospitals [23, 36, 39], and 3 from community hospitals [3, 26, 28].

### 3.1. Clinical Assessment Tools

Different questionnaires were proposed to the patients in 23 studies, by clinical, phone, or postal interview, which are listed in Table 1. Patient's appraisal was done by clinical evaluation during the follow-up visit in 7 studies, while one investigation was based on the review of VA clinical database of the outpatients clinics. 

Patients satisfaction was specifically investigated in 15 papers.

### 3.2. Satisfaction, Quality of Life, and Clinical Symptoms

The mean percentage of patients satisfied by surgery was high (88.9% ± 2.8%).

Ten studies assessed the quality of life after surgery, either comparing it to preoperative values or to a group of medically treated patients. The results are showed in Table 2. Quality of life scores improved after surgery but in only one study out of 4 the surgical group achieved a significantly better score than the medical group.

GERD symptoms scores showed an improvement after surgery in all series. However, GERD-related symptoms (heartburn and/or regurgitation) were still reported in 18.2 ±12.3% of patients (range 4–47%) in 21 studies. Not GERD-related symptoms (including dysphagia, often a new symptom after surgery) were reported in 27.7 ± 18.8%, in 14 papers.

### 3.3. Antireflux Medications

ARMs for GERD-related symptoms after LARS are taken by 34.9% ± 15.9 of patients, in 21 (62.5%) studies (Table 3). Only 6 studies (18.7%) differentiated continuous from occasional treatment, and only 3 studies indicated the main prescriber (GP, gastroenterologist, surgeon, self-prescription). Moreover, only 5 studies indicated the rate of successful response to ARM for GERD-related symptoms (ranging from 25 to 95%), and only one gave details about the response to medical treatment for not GERD-related symptoms.

### 3.4. Endoscopy

The results of endoscopic examination as a part of the clinical assessment after surgery were reported in 8 studies (26.6%).

### 3.5. Esophageal Manometry

This test, specifically performed in 7 studies [8, 11, 15, 17, 19, 20, 38], showed a significant increase of lower esophageal sphincter (LES) pressure after surgery both in symptomatic and asymptomatic patients [19], usually at a short-term evaluation, with a drop in the long term in some studies [20] but not in others [11]. Some [15] did not observe any predictable change of esophageal peristalsis, while disordered esophageal motility was reported in 9% of the patients [19] (no mention of preoperative features). In one study [38] it has been found that patients with either a low or high postoperative LES pressure have a similar long-term symptoms profile with a significant linear correlation between difference in postoperative LES pressure and long-term symptom score for heartburn, dysphagia, and regurgitation. Finally, no correlation has been found between postoperative LES and symptoms or 24-hour pH recording [17].

### 3.6. 24 Hours Esophageal pH-metry

Patients were submitted to esophageal 24-hour pH-metry after LARS in 18 (54.5%) studies, with different indications and results (Table 4). The mean percentage of patients with abnormal score was 24% (range 16–62%), but the percentage of patients submitted to this test was very variable, ranging from 16 to 100%. The mean percentage of abnormal results among those taking ARM was 32% (Table 5).

## 4. Discussion

Laparoscopic antireflux surgery currently represents the golden standard in the surgical management of GERD, being a viable alternative to medical treatment, with minimal morbidity and mortality [8–10]. However, an accurate and universally accepted evaluation of the clinical outcome after LARS is still a critical issue. How to assess satisfaction and subjective symptoms of the patients, how and when to evaluate objectively the outcome in order to define an optimal response to surgery, and, finally, the connotation of a treatment failure, are still controversial topics. The surgical reports analyzed may be divided in 4 different groups:

- papers concentrated on perioperative morbidity and mortality or on technical problems, that is, type of fundoplication and their side effects and less deeply focused on a clear-cut long-term appraisal of the clinical outcome;
- papers dealing with long-term symptomatic results, taking into account symptoms score, quality of life, and patient's satisfaction;
- papers highlightening the large number of patients taking ARM after LARS, generally prescribed on the base of the false assumption that foregut symptoms in a patient after fundoplication are consequent to a failed operation;
- papers concentrated on the comparison of clinical outcome between medically and surgically treated patient population.

Clearly, this paper has inherent limits: the subjective choice of the reports to evaluate and the fact that it is neither a meta-analysis nor a systematic review of the whole literature. It mirrors, however, the current practice. Moreover, while most papers examined are coming from specialized and high-volume surgical centers for LARS, others report the result from community hospitals [28] or from cooperative studies in hospitals at various levels of experience [6, 23]. It is well known that surgeon's experience does matter and that outcomes of laparoscopic fundoplication in routine clinical practice are poorer than those reported by referral centres [3]. It was therefore not in the aims of this study to evaluate surgical results but rather to compare and highlight the difference/homogeneity of postoperative evaluations and to assess their clinical relevance.

### 4.1. Clinical Assessment

Only paper [8] specifically indicate a clinical interview as a part of the evaluation of postoperative symptoms. Most studies relied on mailed questionnaire or phone interview or even on the administrative and clinical database of outpatients clinics. Four different symptoms scores were used. All have been someway validated for clinical practice, but this disparity in the analysis tools certainly reveals a rather unstandardized approach to symptoms' evaluation. Moreover, the way the information is collected as well as the completeness of the followup, sometimes very low, may influence the results and may account for some apparent differences in the clinical outcome, with a wide variation of the typical GERD-related symptoms (i.e., heartburn and/or regurgitation), ranging from 4.8 to 30% amongst the papers examined. Studies relying solely on mailed questionnaires may falsely elevate success rates, especially if followup is incomplete, and patients with worse outcomes may not be motivated to return the questionnaires [41]. In addition, outcomes reported at telephone interview may be more favorable, as well as there is a significant risk of bias in reporting of surgical outcomes when incomplete data are analyzed. The limitation inherent to outcome's comparison between different groups applying different data collection has already been outlined [41] and recently it has been strongly recommended the development of validated outcome instruments [42, 43]. 

The relevance of the presence of symptoms in the evaluation of clinical outcome may also be questioned, being often independent on an objective evidence of persistent GE reflux [44]. Symptomatic assessment has been shown to have low sensitivity and low positive predictive value for abnormal postoperative 24-hour pH-metry. Hence, it might be misleading to report a successful outcome after LARS, relying mainly on symptoms, whose sole presence is a poor indicator of recurrent reflux disease. 

Assessment of quality of life has also been employed as outcome measure after antireflux surgery. In this study, we found that six different questionnaires were used to analyze the QoL, showing again a lack of homogeneity and standardization. In spite of this, results are consistent, and quality of life seems to improve uniformly after surgery in all reports, even in the long term, achieving the same scores observed in a normal sample population or in the group of medically treated patients. Although symptomatic (heartburn) patients, with or without a positive pH study, did not show any different quality of life score after surgery [40], this parameter measured by a validated (and uniform) survey instrument could perhaps be as important as objective measurements of the esophageal function in assessing the clinical status of the patients after LARS.

The percentage of patients satisfied with surgery was generally high. Satisfaction is clearly linked to the improvement or not of quality of life and of course to the presence/absence of symptoms, severe symptoms being usually associated to a significant decrease in patient's satisfaction [32]. It is worthwhile mentioning that in a study [3], the satisfaction rate in patients without resolution of the symptoms was 69%. The use of ARMs does not influence significantly the satisfaction rate, thus suggesting that often the indications for these drugs are for vague and nonspecific symptoms, together with a low threshold by the patients for reinitiating medical treatment. In reality, a high proportion of patients, who complain moderate symptoms or side effects following surgery or who still require regular medication, are of the opinion that a fundoplication was to some extent advantageous. It comes out that relying on satisfaction only for a successful clinical outcome may be ambiguous and that it is needed a clear-cut definition or uniform score for satisfaction, a parameter which may reward the surgeon but cannot probably be taken as a precise and reliable index of a successful LARS.

### 4.2. Antireflux Medications

One third of the patients is taking ARM after LARS in our review, but only 6 studies precise if the use of ARMs was regular or occasional [3, 4, 22, 23, 27, 36]. A recent meta-analysis of RCTs [45] found that—after antireflux surgery—14% of patients still require ARMs. This figure increases with the duration of followup, and up to one third of patients required acid-lowering drugs after 10 years. The data from nonrandomized studies [46], which are higher than data from randomized studies (i.e., 20% of patients under ARMs), are probably more representative of the current clinical practice.

Some authors consider medication use as an outcome measure for successful antireflux surgery [6], while others suggest that use of ARM does not correlate with true recurrent reflux in the majority of the patients [18, 20, 32] and does not necessarily indicate a failure of the procedure. A significant proportion of patients taking medications after operation are using them to relieve nonreflux-related symptoms, and only one third of patients of them showed an abnormal exposure to acid (Table 5). In one study, 79% of patients on ARM took drugs for abdominal or chest symptoms thought to be unrelated to reflux, often pre-existing to surgery [2]. Many of these patients may restart medications on their own or have them prescribed empirically without proven needs. An analysis of an administrative database, likely addressed to patients receiving care from the usual caregivers than from expert providers, highlights the likelihood of continued antireflux medications after surgery in up to 50% of patients [26]. Therefore, not only the high postoperative use of ARM is questionable and often incorrect, but also it may not be a reliable and trustworthy tool for the evaluation of surgical outcome.

### 4.3. Objective Evaluation of the Esophagus

In general, objective outcome measures, probably the better way to evaluate the outcome, are not used frequently, especially in the long-term followup, due to the difficulty of the patients to accept uncomfortable procedures, and this consequently brings a less complete followup. Usually, postoperative objective testing is recommended in presence of persistent or recurrent symptoms after LARS and not in asymptomatic patients, which is realistic in an era of cost containment. However, this approach may not be appropriate, since many symptomatic patients do not show any pathologic reflux at 24 pH-metry; conversely, asymptomatic patient may have significant pathological reflux [19]. 

### 4.4. Endoscopy

Upper GI endoscopy was carried out in a low percentage of patient's population and failed to provide any useful critical information. Relationship with symptoms was poor, and the evaluation and grading of esophageal lesions (when present) were found to be extremely subjective. As a consequence, “standard” endoscopic examination is unlikely to influence postoperative management.

### 4.5. Esophageal Manometry

While a significant postoperative increase of LES pressure has been found in successful, asymptomatic patients [47], other investigations failed to show any significant difference in pressure increase between symptomatic and asymptomatic patients [48]. Moreover, no correlation has been found between postoperative LES pressure and symptoms or 24 hour pH-metry results [17]. Taking into account the inconsistent manometric findings and the difficult acceptance of the procedure by the patients, it is hard to propose it as a regular and trustable postoperative test, its role being secondary to esophageal pH recording in symptomatic patients.

### 4.6. 24 Hour Esophageal pH-metry

In the papers examined, postoperative 24-hour pH-metry has been the most frequently performed objective test, mainly to identify patients with true recurrent gastroesophageal reflux. The reproducibility of 24-hour pH monitoring is essential to make it reliable. Actually, a concordance rate of 96% in repeated test was recently reported [40]. Ideally, patients with recurring symptoms should undergo a 24-hour pH probe study for an objective evaluation and quantitation of acidic reflux. We do not feel that such test should be recommended postoperatively on a routine basis. Indeed, finding a positive test in an asymptomatic patient would be challenging due to the lack of established guidelines in this clinical setting. On the other hand, successful operations, as demonstrated by a normal exposure to acid, may not eliminate all reflux-related symptoms. Although nonacid reflux could be responsible for symptoms, it has been shown to be very uncommon [49]. Moreover, a recent investigation [50] reported that persistent symptoms are neither caused by acid nor by weakly acidic reflux, but rather by abnormal air handling. To investigate weakly acidic or nonacidic reflux-related symptoms, a combined pH-impedance study is needed, but this test is more costly and technically demanding.

## 5. Conclusions

The evaluation of efficacy of LARS as a permanent treatment for GERD definitely depends on determining what should be considered a successful outcome. This study highlights the need to be careful when considering clinical outcomes reported after antireflux surgery. The complexity in capturing data is evident. Not only symptoms assessment may be considered not appropriate in some studies, but also symptoms scores and outcome variables reported in different studies are dissimilar, making a plea for more uniform symptoms scales and quality of life tools. This would be of utmost importance in the clinical practice, where either gastroenterologists or primary care physicians need to understand that most patients complaining of postoperative symptoms do not have pathologic reflux. 

Relying on patient's satisfaction to define a successful surgical outcome may be ambiguous and cannot probably be taken as a precise and reliable index of a successful procedure, yet from this study it can be considered a practical and simple tool, with uniform results.

## Figures and Tables

**Table 1:** Parameters used for patients evaluation and number of studies.

Parameters	Number of studies	Refs
Questionnaires		
GERD symptoms score	23	[3–6, 8, 11, 15, 16, 18, 20, 24, 25, 27, 29, 32–37, 39, 40]
Gastroesophageal Reflux Disease Activity Index (GRACY)		
Digestive Health Survey Instrument		
Gastrointestinal Symptoms Rating Scale (GSRS)		
Jamieson Score		
Gastrointestinal Quality of Life Index (GIQLI)	3	[25, 27, 33]
Psychological General Well-Being Index (PGWBI)	2	[23, 39]
Short-form 36 (SF 36)	2	[6, 22]
Health-Related Quality of Life (HRQL)	1	[34]
Well-Being Score (WBS)	1	[2]
Visual Analogue Score (VAS)	1	[11]
Patients satisfaction	16	[2–6, 11, 18, 20, 24, 29, 32–35, 38]
VA clinical data base (outpatients clinics)	1	[26]
Clinical assessment at follow-up visit	7	[4, 11, 19, 22, 31, 38, 39]
Objective esophageal tests		
Endoscopy	8	[3, 6, 14, 15, 22, 23, 30, 31]
Esophageal manometry	7	[8, 11, 15, 17, 19, 20, 38]
24-h Esophageal pH-metry	18	[6, 8, 11, 14–20, 27, 32–37, 40]

**Table 2:** HRQoL Assessment with different questionnaires and their results.

Author	Year	Ref.	Tools	Results
Spechler et al.	2001	[6]	SF-36	No significant difference between medical and surgical group
Bammer et al.	2001	[2]	WBS	Improved significantly at more than 5 years of followup
Olberg et al.	2005	[23]	PGWBI	No significant difference between medical and surgical group
Contini and Scarpignato	2004	[22]	SF-36	Normal score 2 years after LARS
Ciovica et al.	2006	[27]	GIQLI + HRQL	QoL normalized after LARS and significantly higher than a medically treated group
Dallemagne et al.	2006	[25]	GIQLI	Significantly better than preoperatively at 10 years
Draaisma et al.	2006	[11]	VAS	30% improvement after surgery
Gee et al.	2008	[34]	GERD−HRQL	Near normal score at long-term followup
Fein et al.	2008	[33]	GIQLI	Significant improvement of the QoL after 10 years
Lundell et al.	2009	[39]	PGWBI	Similar to that of normal population in both surgical and medical group at 12 years

VAS: Visual Analogue Scale.

**Table 3:** Incidence of ARM use after LARS.

Authors	Year	Ref.	Followup (yrs)	Patients on ARM (%)
Spechler et al.	2001	[6]	6.4	23/37 (62%)
Bammer et al.	2001	[2]	6.3	24/171 (14%)
Booth et al.	2002	[18]	2.0	19/140 (14%)
Lord et al.	2002	[14]	2.4	37/86 (43%)
Anvari and Allen	2003	[20]	5.0	21/181 (12%)
Bloomston et al.	2003	[5]	5.0	31/84 (37%)
Papasavas et al.	2003	[8]	2.6	56/297 (19%)
Vakil et al.	2003	[3]	1.7	26/80 (33%)
Velanovich et al.	2003	[21]	2.4	16/122 (13%)
Galvani et al.	2003	[15]	1.5	62/124 (50%)
Contini and Scarpignato	2004	[22]	2	13/50 (26%)
Tucker et al.	2005	[24]	4.1	58/119 (49%)
Thibault et al.	2006	[28]	3.6	38/121 (31%)
Dominitz et al.	2006	[26]	4.5	1199/2406 (49.8%)
Draaisma	2006	[11]	6	11/79 (13.9%)
Bonatti et al.	2007	[4]	2.4	37/94 (39%)
Zaninotto et al.	2007	[31]	8.0	30/145 (21%)
Wijnhoven et al.	2008	[36]	5.9	312/844 (37%)
Oelschlager et al.	2008	[35]	5.7	119/288 (41%)
Thompson et al.	2009	[40]	4.6	42/69 (60.8%)
Lundell et al.	2009	[39]	12	55/144 (38%)

				Mean = 34.9%

**Table 4:** Postoperative 24 hrs pH-metry. Indications and results.

Authors	Year	Ref.	Pts submitted to pH-metry (%)	Indications	Results
Franzén et al.	1999	[16]	67/101 (66.3%)	Follow-up	19.4% abnormal score
Spechler et al.	2001	[6]	10/37 (27%)	Follow-up	No statistical difference between medical and surgical group. Small sample. Results inconclusive.
Lord et al.	2002	[14]	86/86 (100%)	Symptomatic patients after LARS	23% abnormal score
Arca et al.	2002	[17]	28/46 (49%)	Follow-up	28% abnormal score
Booth et al.	2002	[18]	109/175 (78%)	Follow-up	5% abnormal score
Khajanchee et al.	2002	[19]	209/209 (100%)	Follow-up	16.7% abnormal score
Galvani et al.	2003	[15]	124/124 (100%)	Symptomatic patients after LARS	39% abnormal score;
Gee et al.	2008	[34]	20/191 (10.4%)	Follow-up (ARM)	70% abnormal result
Anvari and Allen	2003	[20]	181/332 (54.5%)	Follow-up	Mean acid reflux score significantly lower than preop
Papasavas et al.	2003	[8]	93/297 (31.3%)	Follow-up	Average percentage of exposed time <4 was significantly reduced
Ciovica et al.	2006	[27]	351/351 (100%)	Follow-up	10% Abnormal score
Draaisma et al.	2006	[11]	?/79	Follow-up	12.5% Abnormal score
Morgenthal et al.	2007	[32]	/166	Follow-up	14% abnormal score in pts on ARM (3/21)
Oelschlager et al.	2008	[35]	58/288 (20.1%)	Follow-up (heartburn)	22% abnormal result
Wijnhoven et al.	2008	[36]	139/844 (16.4%)	Patients taking ARM after LARS	22.3% abnormal results
Boddy et al.	2008	[37]	106/145 (73.1%)	Follow-up (4 months)	No correlation between pH scores and symptoms score
Fein et al.	2008	[33]	67/99 (67.6%)	Follow-up	33% of pts with heartburn had recurrent reflux
Thompson et al.	2009	[40]	69/69 (100%)	Symptomatic patients after LARS	22% Abnormal score

**Table 5:** Abnormal esophageal exposure to acid in patients taking ARM after LARS.

Authors	Year	Ref.	Followup (months)	Pts with abnormal pH score (%)
Booth et al.	2002	[18]	24	7/19 (36.8%)
Lord et al.	2002	[14]	27.8	9/37 (24.3%)
Galvani et al.	2003	[15]	17	48/124 (39.0%)
Anvari and Allen	2003	[20]	60	9/21 (42.8%)
Draaisma et al.	2006	[11]	60	Absence of correlation between the use of PPIs and documented reflux symptoms
Thompson et al.	2009	[40]	44	17/53 (32%)
Wijnhoven et al.	2008	[36]	70.8	16/61 (26.2%)
Fein et al.	2008	[33]	24	NA (42%)
Thompson et al.	2009	[40]	55	7/42 (16.6%)
				Mean = 32.4%
